# Multi-scale comparative transcriptome analysis reveals key genes and metabolic reprogramming processes associated with oil palm fruit abscission

**DOI:** 10.1186/s12870-021-02874-1

**Published:** 2021-02-11

**Authors:** Kim Fooyontphanich, Fabienne Morcillo, Thierry Joët, Stéphane Dussert, Julien Serret, Myriam Collin, Philippe Amblard, Sithichoke Tangphatsornruang, Peerapat Roongsattham, Chatchawan Jantasuriyarat, Jean-Luc Verdeil, Timothy J. Tranbarger

**Affiliations:** 1 UMR DIADE, Institut de Recherche Pour le Développement, Université de Montpellier, IRD Centre de Montpellier, 911 Avenue Agropolis BP 64501, 34394 Cedex 5 Montpellier, France; 2 Grow A Green Co, Ltd. 556 Maha Chakraphat Rd. Namaung, Chachoengsao, Chachoengsao Province 24000 Thailand; 3 CIRAD, DIADE, F-34398 Montpellier, France; 4 PalmElit SAS, Montferrier-sur-Lez, France; 5 National Science and Technology Development Agency, 111 Thailand Science Park, Phahonyothin Road, Pathum Thani, Thailand; 6 Department of Genetics, Faculty of Science, Kasetsart University Bangkhen Campus, 50 Phahonyothin Road Jatujak, Bangkok, Thailand; 7 CIRAD, UMR AGAP, F-34398 Montpellier, France; 8 AGAP, Univ Montpellier, CIRAD, INRAE, Institut Agro, Montpellier, France

**Keywords:** Transcriptome, Fruit abscission, Abscission zone, Monocotyledon, Metabolic reprogramming, *Elaeis guineensis*

## Abstract

**Background:**

Fruit abscission depends on cell separation that occurs within specialized cell layers that constitute an abscission zone (AZ). To determine the mechanisms of fleshy fruit abscission of the monocot oil palm (*Elaeis guineensis* Jacq*.*) compared with other abscission systems, we performed multi-scale comparative transcriptome analyses on fruit targeting the developing primary AZ and adjacent tissues.

**Results:**

Combining between-tissue developmental comparisons with exogenous ethylene treatments, and naturally occurring abscission in the field, RNAseq analysis revealed a robust core set of 168 genes with differentially regulated expression, spatially associated with the ripe fruit AZ, and temporally restricted to the abscission timing. The expression of a set of candidate genes was validated by qRT-PCR in the fruit AZ of a natural oil palm variant with blocked fruit abscission, which provides evidence for their functions during abscission. Our results substantiate the conservation of gene function between dicot dry fruit dehiscence and monocot fleshy fruit abscission. The study also revealed major metabolic transitions occur in the AZ during abscission, including key senescence marker genes and transcriptional regulators, in addition to genes involved in nutrient recycling and reallocation, alternative routes for energy supply and adaptation to oxidative stress.

**Conclusions:**

The study provides the first reference transcriptome of a monocot fleshy fruit abscission zone and provides insight into the mechanisms underlying abscission by identifying key genes with functional roles and processes, including metabolic transitions, cell wall modifications, signalling, stress adaptations and transcriptional regulation, that occur during ripe fruit abscission of the monocot oil palm. The transcriptome data comprises an original reference and resource useful towards understanding the evolutionary basis of this fundamental plant process.

**Supplementary Information:**

The online version contains supplementary material available at 10.1186/s12870-021-02874-1.

## Background

Fruit abscission coordinates seed dispersal, which is essential for plant reproductive success. In an ecological setting, fruit that shed prematurely before seeds are fully developed, or too late during seasonal climate changes can jeopardize reproductive success of wild species. Fruit detachment mechanisms have also been a target of domestication, and a well-known domestication example is the non-abscising *jointless* mutant, which has been used to develop non-shedding tomato fruit cultivars [1]. This is also the case with cereals (rice, barley wheat, etc.) for which domestication has allowed the selection of varieties with non-shedding grains.

Indeed, fleshy fruit abscission is an important agronomic trait with widespread economic consequences [2]. Fruit bearing species with overly abundant immature fruit need to be thinned to obtain the optimal size and highest quality fruit. Crops with immature fruit that shed prematurely need to be inhibited to allow appropriate yields, while mature fruit that shed too soon need to be inhibited to facilitate harvest and avoid economic losses [3]. Whereas fruit abscission is conceptionally simple (i.e. the fruit separates from the plant and falls), the cell separation and regulatory mechanisms that allow fruit to be detached and shed involve highly spatially, temporally and environmentally regulated molecular and biochemical processes. Interactions between multiple signalling pathways that integrate the environment and the overall developmental and physiological status of the plant are necessary for fruit abscission to occur [4–9].

As with other plant organ abscission phenomenon, fruit abscission occurs through the function of a specialized tissue referred to as the abscission zone (AZ), located at the base of the organ to be shed, where breakdown of cell-to-cell adhesion occurs that results in cell separation and organ detachment [10]. The *jointless* tomato, which lacks a functional AZ, exemplifies the central role of the AZ for the abscission process [1]. Abscission zones can be classified into two general types based on their anatomy: 1) at the boundary region at the base of the organ to be shed and the neighbouring tissue, for example between a floral organ and the pedicel as observed in *Arabidopsis* or between the mesocarp and the pedicel as observed in oil palm fruit, and 2) within a tissue as observed in the pedicel of the tomato fruit or flower [2, 4]. While the differentiation of these two AZ types may be different, in both cases once the AZ develops at the base of the organ to be shed, they must acquire competence to respond to signals required for cell separation and organ abscission [4, 6, 11–14]. After the AZ becomes competent for separation to be induced, cellular activity, in particular the expansion of the golgi vesicles and activation of the endomembrane system with the release of hydrolytic enzymes to the apoplast, leads to the degradation of the middle lamella and ultimately cell separation, and the organ is shed [3, 15, 16].

Currently, the main model of monocot fleshy fruit abscission is from research on ripe fruit abscission of oil palm (*Elaeis guineensis*) of the palm family (Arecaceae). The oil palm fruit primary AZ is located at the junction between the mesocarp and the pedicel, and is a large multi-cell layer AZ, with minor adjacent AZs that separate later after separation in the primary AZ is complete [17, 18]. Common to organ abscission in many species, ethylene or its precursor 1-aminocyclopropane-l-carboxylic acid (ACC) promotes, while auxin inhibits, cell separation in the primary AZ of the oil palm fruit [19, 20]. Ethylene production is initiated in the apex of the ripe fruit mesocarp and progresses to the base of the mesocarp in a positive correlation to the percentage of separation that occurs in the primary AZ, which suggests ethylene may serve to link fruit ripening to fruit abscission [19]. The precise position of separation in the smaller adjacent AZs is determined by the age and ripeness of the fruit and depends upon completion of separation in the primary AZ [17]. In addition, while the vascular strands between the fruit base and the pedicel are continuous across the primary AZ, they are much less lignified and may facilitate organ removal [17].

The oil palm fruit primary AZ cells have high amounts of unmethylated pectin in the cell wall, in addition to increased polygalacturonase (PG) enzyme activity during abscission [21], which is also observed during fruit abscission of many species [22]. A further decrease in methylesterified homogalacturonan (HG), the most abundant pectin polymer, is detected particularly in AZ cells where cell separation occurs [23]. An oil palm fruit PG transcript (*EgPG4*) is abundant in the AZ in response to ethylene, and preferentially increased in the AZ cell layers prior to cell separation [20]. However, *EgPG4* is also expressed in the mesocarp during ripening and is not specific to the AZ, and may function to dismantle the pectin rich cell walls in both tissues [20, 24]. Based on these results, while both demethylesterification and hydrolysis of the pectin HG appear to be important for cell separation in the AZ, it is unclear what other mechanisms are involved for cell separation to occur in the oil palm multi-cell-layer primary AZ.

In the present study, we undertake a large-scale multifaceted analysis of the transcriptional activity in the oil palm ripe fruit AZ induced by ethylene. We compared this expression with that of the neighbouring pedicel tissue treated with ethylene where no cell separation is observed, and with the expression in the AZ of unripe fruit that do not abscise when treated with ethylene. Expression was then validated in ripe fruit undergoing natural abscission under field conditions. Finally, we took advantage of a palm with an unusual non-abscission phenotype to validate the expression and function of selected candidate genes in the AZs of ripe fruit that do not undergo abscission [25]. Our approach allows an integrated view of the transcriptional activities, cellular processes and specificities that underlie the fruit abscission process in this monocot species.

## Results

### Identification of AZ-specific ethylene-responsive genes during ripe fruit abscission using multi-scale comparative transcriptome analysis

**Fig. 1:**
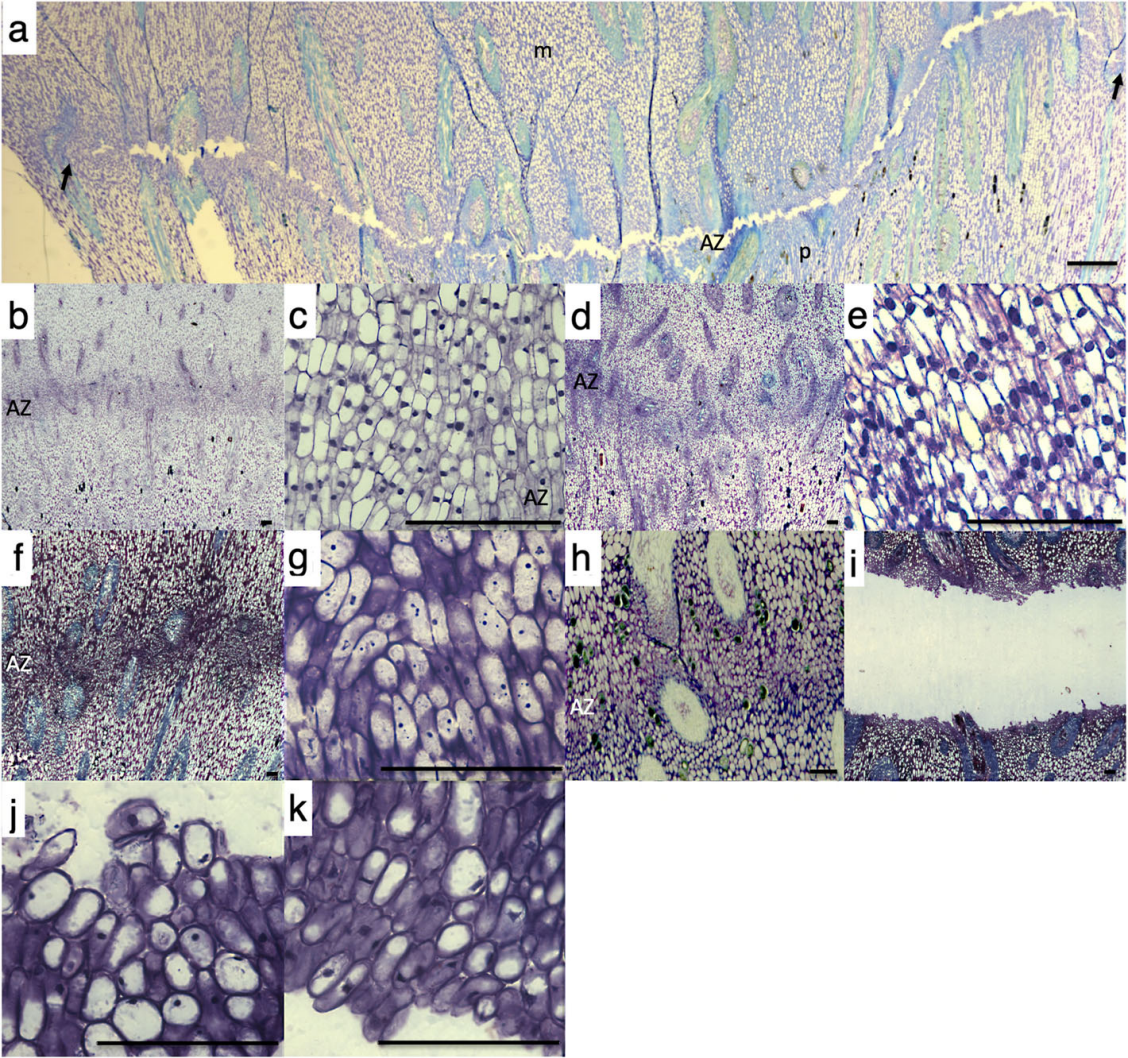
Comparison of the abscission zone, mesocarp and pedicel tissues at the base of the oil palm fruit in response to ethylene and during natural abscission in the field. The mesocarp (m) is above and the pedicel (p) is below the abscission zone (AZ) in all photos. **a** cell separation occurs in the ripe fruit (150 DAP) primary AZ after 9 h of ethylene treatment, but only to a limited extent in the adjacent AZs which are difficult to discern (black arrows). **b** and **c** no cell separation is observed in the tissues (pedicel, mesocarp and AZ), at the base of 30 DAP fruit in the field, or treated with ethylene for 9 h (**d** and **e**). **c** and **e**, Higher magnification view of AZ from 30 DAP fruit in the field and treated with ethylene for 9 h respectively. **f** and **g** No cell separation is observed in the tissues (pedicel, mesocarp and AZ) at the base of 120 DAP fruit in the field. **h** 160 DAP AZ prior to cell separation and after cell separation (**i**) that occurred during natural abscission in the field. **j** Higher magnification view of 160 DAP fruit AZ cells separated on pedicel and on the mesocarp side (**k**) of AZ that underwent natural abscission in the field. Scale bars are 100 μm

**Fig. 2:**
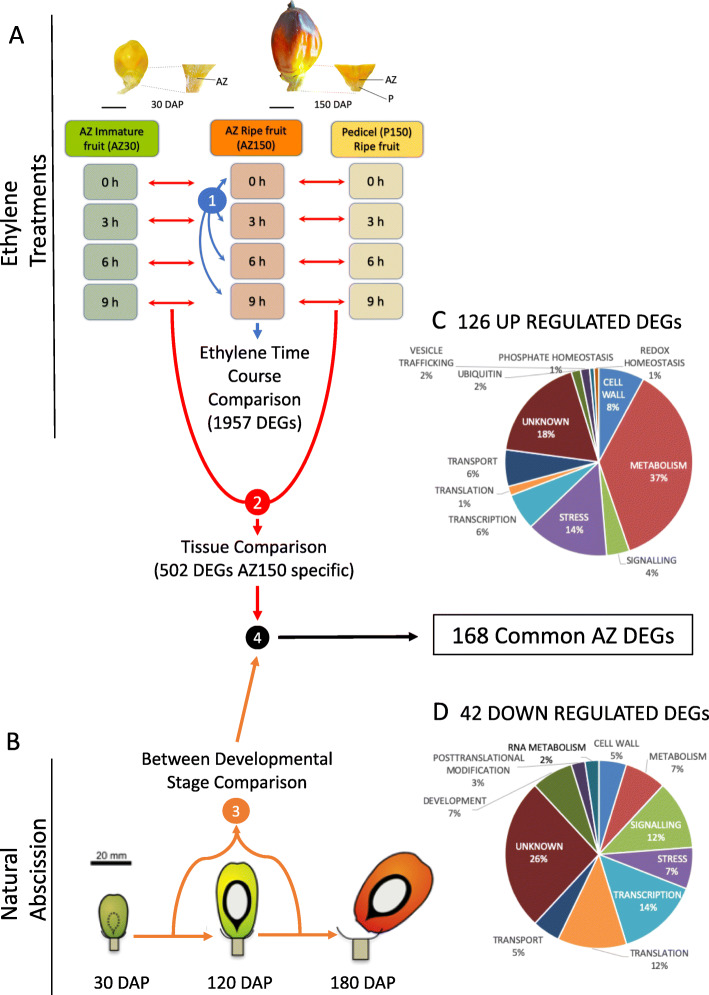
A multi-scale comparative transcriptome analysis approach was used to screen for genes activated or repressed in the ripe fruit AZ during abscission. **a** We first used ethylene to induce abscission in ripe fruit, and compared the expression profiles of the ripe fruit AZ (AZ150) with that of adjacent pedicel tissue (P150) and immature fruit AZ (AZ30), in which no cell separation occurs under ethylene treatments. To screen for preferential ripe fruit AZ expression, differential expression in the AZ150 was first identified (blue arrows, screen step 1), then DEGs with similar profiles in the AZ30 and P150 were eliminated (red arrows, screen step 2). Candidate DEGs were retained when there was a statistical difference between their expression in the AZ150 DAP samples compared with the P150 DAP and AZ30 DAP for at least one time point (0 h, 3 h, 6 h or 9 h). **b** A transcriptome developmental time course (30, 120 and 160 DAP) of the AZ from fruit during natural abscission was then obtained (step 3) to provide a comparison with the DEGs found in the AZ during ethylene induced abscission (screen step 4) that resulted in the identification of 168 DEGs common to both ethylene induced and natural abscission (black arrow). **c** From these DEGs, 126 were up regulated and (**d**) 42 were down regulated in the AZ during abscission in both contexts

As a first step to screen for genes involved in oil palm ripe fruit abscission, we examined the short-term gene expression response in the fruit AZ at 150 Days after pollination (DAP) to an exogenous ethylene treatment. Transcriptome analysis was assessed every 3 h over a period of 12 h, while cell separation was already observed after 9 h of treatment (Fig. 1a). We identified 1957 differentially expressed genes (DEGs) during at least one time point of the ethylene treatment in the 150 DAP sample (AZ150) compared to the control (time 0 h AZ150; Fig. 2a; Supplementary Table 1). Hierarchical clustering analysis identified 4 clusters of DEGs with different sub-clusters A1-A7, B1-B6, C1-C7, D1-D10 (Supplementary Fig. 1). Cluster A includes genes with a continuous increase of expression under ethylene treatment, while cluster D gathers genes down-regulated by ethylene. Genes belonging to clusters B and C display short-term responses to ethylene with maximal expression observed at 3 to 6 h respectively after initiation of exogenous ethylene application.

As a second step to screen the 1957 DEGs for AZ specific expression, we compared the short-term response of AZ150 to exogenous ethylene with those from the AZ from immature fruit at 30 DAP (AZ30; Fig. 1b-e) and the pedicel from ripe fruit (P150; Fig. 1a) samples (also treated with ethylene at same time intervals) at each ethylene treatment time point (Fig. 2a). From this analysis, a total of 502 DEG candidates were retained based on their higher or lower expression level in AZ150 compared to that observed in both AZ30 and P150 (Supplementary Table 2).

**Fig. 3:**
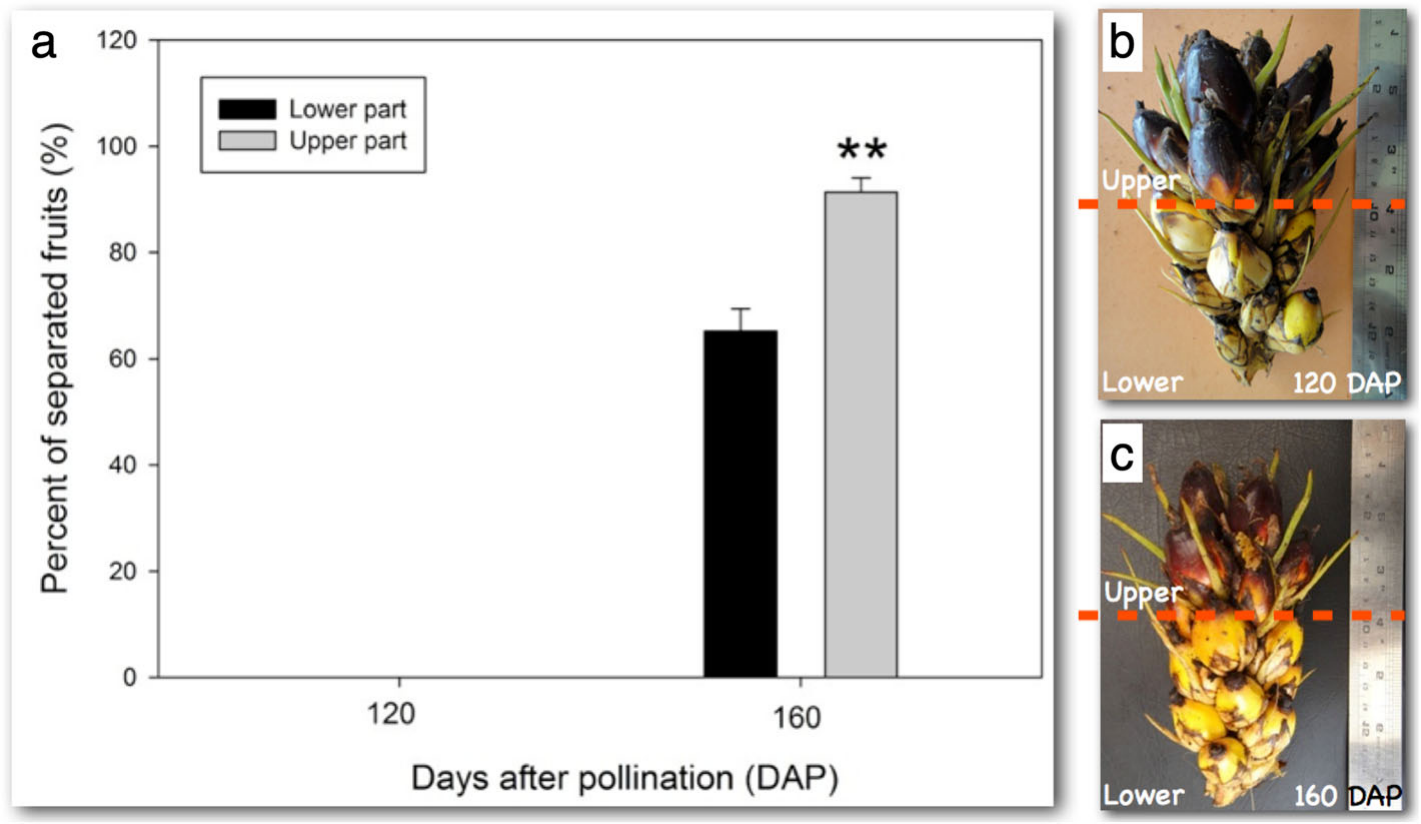
Only ripe fruit in the field undergo natural abscission and ripening is unsynchronized within the spiketlet. **a** A test for loose fruit was performed to compare developing (**b**, 120 DAP) and ripe fruit (**c**, 160 DAP) in the field. The fruit on the spikelets were tested for looseness by pulling on the individual fruit. No loose fruit were found at 120 DAP, while loosened fruit were found at 160 DAP. While no difference was found within the spikelet for fruit at 120 DAP, the fruit from the upper half of the ripe fruit spikelet at 160 DAP separated more than the lower half. The expression profiles of the DEGs candidates was performed with the AZ from the fruit at 120 DAP when no loosening was observed and compared with the AZ from fruit where loosening was observed (160 DAP)

As a third step to screen for AZ genes involved in abscission, we analysed gene expression in the AZ of ripe fruit undergoing natural abscission in the field and aimed at finding genes with similar expression profiles between ethylene induced and natural abscission in the field (Fig. 2b). The study was conducted with samples from the AZ collected in the field at 30 DAP (Fig. 1b and c), 120 DAP (Fig. 1 f and g), during which time no abscission was observed, and 160 DAP (h-k) collected from a fruit bunch where abscission was observed (Fig. 1 f-k). A physical pull test revealed that none of the fruit from the 120 DAP spikelet could be removed when pulled, while the fruit from the lower (65%) and upper portion (92%) of the 160 DAP spikelet were easily removed and the fruit separated along the primary AZ (Fig. 3a, b and c). In addition, tissue samples were taken and confirmed that separation only took place in the AZ from fruit at 160 DAP spikelets, while no separation is observed in the AZ at 30 or 120 DAP (Fig. 1b, c, f, g, i-k).

A total of 168 DEGs were found to have expression profiles comparable during both ethylene induced and natural field abscission, including 126 up-regulated and 42 down-regulated genes in the AZ during abscission, and were retained for further analysis of processes and genes associated with abscission (Fig. 2c and d; Supplementary Table 3). Among the 168 DEGs, 34 had unknown annotations, while 134 candidate genes were assigned putative functions, and the most-represented functional classes included metabolism (49), followed by stress (21), transcription (14), cell wall (12) and signalling (10).

### Validation of abscission-related genes through a gene expression survey in the AZ of a non-shedding oil palm variant

**Fig. 4:**
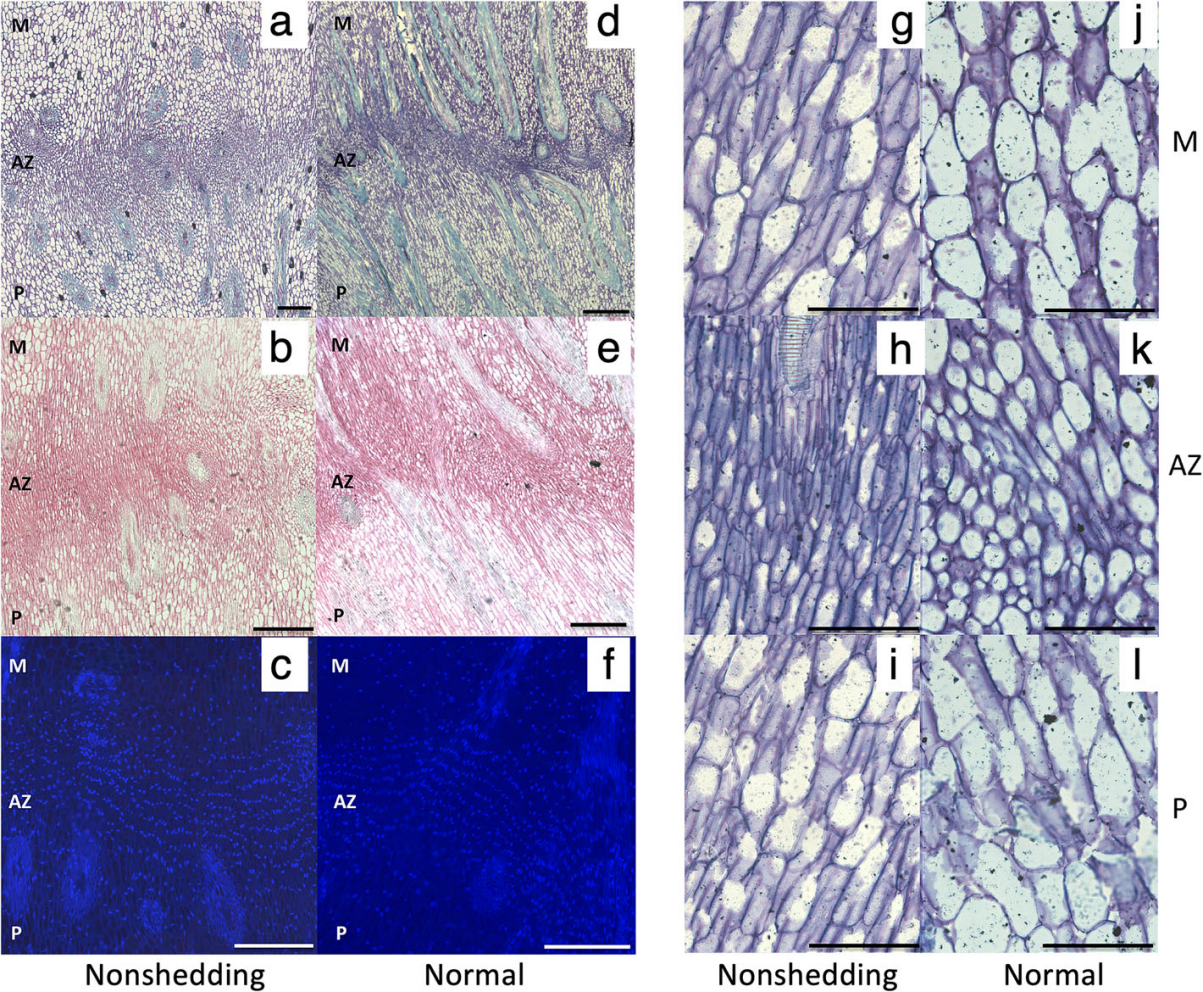
No abnormalities are observed in the non-shedding MTC180 AZ that has characteristics of a functional AZ. A comparison of the fruit AZ of the non-shedding individual MTC180 **(a-c)** with that of a normal functional AZ in ripe fruit at 160 DAP **(d-f)** reveals fully differentiated AZ cell characteristics including aligned nuclei and small cytoplasmically dense cells, and no apparent abnormalities in the non-shedding AZ. Longitudinal sections were stained with toluidine blue (**a, d, g-l**), ruthenium red (**b and e**) and DAPI (**c** and **f**). M, mesocarp; AZ, abscission zone; P, pedicel; Scales bars are 100 μm **(a-f)** and 50 μm for **(g-l)**

**Fig. 5:**
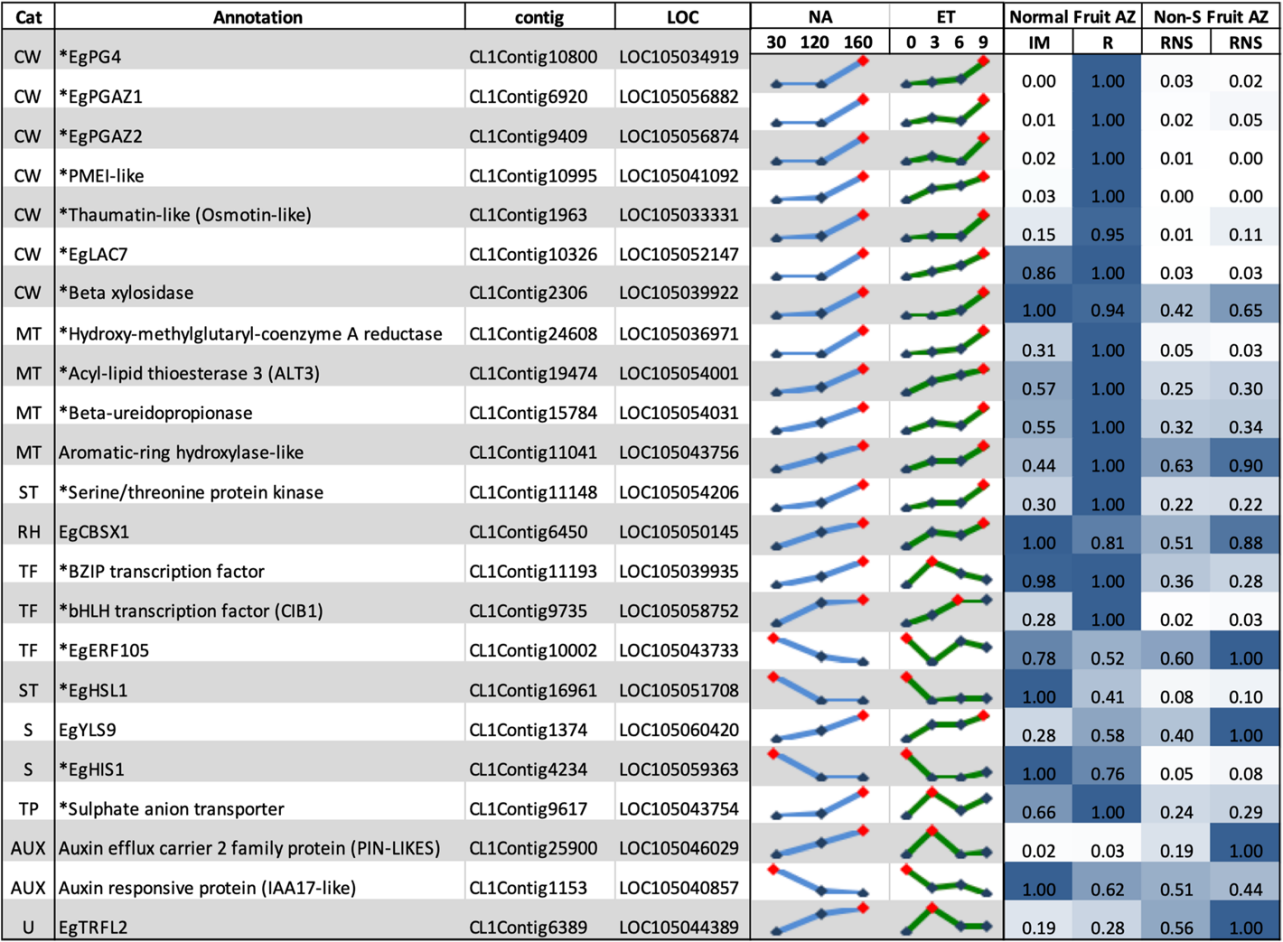
A comparison of the expression profiles of selected gene candidates in the AZ during ethylene induced abscission (ET), natural abscission (NA) that occurs in the field and in the AZ fruit that remain attached to the fruit bunch and are not shed (Non-S). The NA column includes samples from 30, 120 and 160 DAP. The ET column includes profiles of statistically significant candidates expressed preferentially in the AZ during ethylene induced abscission (0, 3 h, 6 h and 9 h of treatment). Expression profiles of many candidates in the AZ during ethylene induced and natural abscission (column ET and NA) are opposite to that found in the non-shedding fruit AZ. Normal backcross (IM and R) eventually shed their fruit, while the non-shedding backcross (RNS columns) do not. Results of the expression profile from qPCR shown by the heat map with the highest peak of expression calculated as 1. CW, Cell Wall; MT, Metabolism; TF, Transcription Factor; AUX, auxin related; TP, Membrane Transport; ST, Signal Transduction. IM, Immature Fruit; R, Ripe fruit; RNS, Ripe Non-Shedding fruit)

To provide validation for functional roles of the 168 candidate genes identified to be involved in the abscission process, the expression profiles of a selected subset (23) were examined in the AZ of an individual oil palm (MTC180), previously shown to not shed its fruit [25]. While the AZ of the ripe fruit from MTC180 are differentiated with similar cellular characteristics as with individuals that shed their ripe fruit normally, MTC180 fruit remain on the trees and no natural abscission in the field was observed (Fig. 4 [25];). Our hypothesis was that the non-shedding character of this individual is due to perturbations in the gene expression network required for abscission to occur. We predicted that genes important for abscission would have the altered expression profiles in the non-shedding (MTC180) fruit AZ, compared with those observed in the AZ during natural abscission, and in response to ethylene induced abscission. A qRT-PCR analysis of AZ samples from normally ripening and shedding fruit, in comparison with the AZ from fruit that remain attached and are not shed was performed. The candidates selected all had comparable expression during both ethylene induced abscission and natural fruit abscission in the field, and had a range of annotations, including those related to the cell wall, metabolism, signal transduction, transcriptional regulation, stress, transport, auxin transport and response, redox homeostasis, and one unknown. From the 23 candidates selected, 17 of the candidates (72%) had expression profiles in the AZ of non-shedding fruit that matched our predictions, i.e. altered expression profiles in the non-shedding fruit AZ compared to those observed during ethylene induced and natural abscission (Fig. 5; Supplementary Table 5). It should be noted that all the cell wall related transcript profiles matched our predictions, including those that encode PG4 and PGAZ1, PGAZ2, PMEI-like, Thaumatin-like, LAC7 and beta xylosidase. The transcripts for these proteins were detected at very low amounts in the non-shedding AZ compared with their amount found in AZ during the ethylene induced and natural abscission. Three of the four metabolic gene expression profiles also matched our predictions, including transcripts encoding Hydroxy-methylglutaryl-coenzyme A reductase, Acyl-lipid thioesterase 3 (ALT3) and Beta-ureidopropionase. Two transcription factor transcripts encoding a BZIP and bHLH, were down regulated in the non-shedding AZ, while the transcript for EgERF105 was relatively high in the non-shedding AZ compared to during ethylene induced and natural abscission. Finally, two transcripts encoding signal transduction components including a serine/threonine protein kinase and HSL1, (HAESA-like leucine-rich repeat receptor kinase1), were detected in lower amounts in the non-shedding AZ. Such a high level of congruence further strengthens the potential roles that the 168 candidate genes play in the AZ during the abscission process.

### AZ-specific genes expressed during ethylene induced and naturally ripe fruit abscission include genes involved in cell wall modification and degradation, as well as organ separation

Among the 134 annotated gene candidates, 12 genes encode proteins directly involved in the synthesis, modification or degradation of cell wall polysaccharides (Supplementary Table 3). Quite noticeably, half of them encode proteins related to pectin modification and degradation (EgPG4, EgPGAZ1, EgPGAZ2, EgPMEI, EgBXL2, EgLRX4). Among those, *EgPG4* was previously found to be highly expressed in ripe fruit undergoing natural abscission [20]. The others relate to xyloglucan and mannan degradation (EgBGLC1 and EgMAN7, respectively), phenylpropanoid and lignin biosynthesis (EgLAC7, EgC4H/CYP73A5/REF3), callose biosynthesis (EgGSL1) and cell wall acidification (EgHA2). Other annotation categories had additional candidates closely linked to the cell wall, including A cystathionine beta-synthase (CBS) family protein CBSX-2, a key redox sensor that directly regulates the activation of thioredoxins and regulate enzymes involved in lignin polymerization and cell wall thickening during anther dehiscence in Arabidopsis [26, 27], in addition to the synthesis of cell wall precursors (EgMIPS2) and the synthesis of cell wall bound ferulic acid (EgREF1), which may contribute to the different levels of ferulic acid content between dicotyledon and monocotyledon plants [28].

Strikingly, among those cell-wall related genes, five encode proteins with similarities to those identified with functional roles during Arabidopsis floral organ abscission, anther dehiscence, silique dehiscence or seed abscission including: sequences similar to ADPG1, MANNANASE7 (MAN7), LACCASE7 (LAC7), cystathionine beta-synthase (CBS) family protein (CBSX1) (Table 1 [29–31]. Our candidate gene list also includes a HEASA-like leucine-rich repeat receptor kinase, unrelated to cell-wall metabolism, but shown to be involved in Arabidopsis floral organ abscission [32]. In addition, a BEL1-like transcription, similar to SH5 in rice, which induces grain shattering through the control of abscission-zone development and the inhibition of lignin biosynthesis, was also identified [33]. These findings validate our approach used to identify AZ genes with functional roles during oil palm ripe fruit abscission, and provide evidence for common mechanisms related to cell separation between monocots and dicots.

**Table 1:** DEGs with similarities to genes with functional roles in cell separation events

Contig Name	Eg Locus	TAIR9 accession	Best manual annotation	Gene name	Reference	Separation event
CL1Contig6920	LOC105056882	AT3G57510	Endo-polygalacturonase (ADPG1/PGDZAT)	*EgPGAZ1*	[29, 30]	Arabidopsis anther dehiscence, silique dehiscence, seed abscission
CL1Contig9409	LOC105056874	AT3G57510	Endo-polygalacturonase (ADPG1/PGDZAT)	*EgPGAZ2*	[29, 30]	Arabidopsis anther dehiscence, silique dehiscence, seed abscission
CL1Contig10326	LOC105052147	AT3G09220	Laccase 7	*EgLAC7*	[5]	Arabidopsis floral organ abscission
CL1Contig20303	LOC105040138	AT5G66460	Endo-beta-mannase 7/Glycosyl hydrolase superfamily protein/MANNANASE7	*EgMAN7*	[31]	Arabidopsis silique dehiscence
CL1Contig16961	LOC105051708	AT1G72180	Receptor-like protein kinase HSL1	*EgHSL1*/*CEPR2*	[32]	Arabidopsis floral organ abscission
CL1Contig6450	LOC105050145	AT4G36910	Cystathionine beta-synthase (CBS) family protein	*CBSX1*/*LEJ2*	[26, 27]	Arabidopsis anther dehiscence
CL1Contig17462	LOC105059537	AT5G41410/ Os05g38120	Homeobox protein BEL1 homolog / SH5	*EgBEL1*	[33]	Rice seed shattering

### Hormone pathways are activated in the AZ during abscission

From the comparison between ethylene induced and natural abscission, we observed a number of ethylene related genes with common transcription profiles in both contexts. Genes identified encode proteins involved in ethylene biosynthesis (1-AMINOCYCLOPROPANE-1-CARBOXYLATE OXIDASE5, ACO5), perception (ETHYLENE INSENSITIVE4, EIN4), mobilization of the ethylene precursor 1-aminocyclopropane-1-carboxylic acid (ACC) lysine histidine transporter (LHT1) [34];) and ethylene response factors (ERF18 and RAP2.2), in addition to the transcription factors related to ethylene response including NAC6, MYC2, and EIN3 (Supplementary Table 3). Interestingly in Arabidopsis, the transcription factor NAC6 regulates *ACO5* and cell wall modifying enzyme genes in an ethylene dependent manner, while MYC2 and EIN3 were found to modulate ethylene and jasmonate (JA) activity antagonistically [35, 36]. The genes that encode for ACO5, EIN4, NAC6, MYC2 and RAP2.2 were all upregulated, while those for EIN3 and ERF18 were down regulated during abscission. In addition to ethylene, transcripts related to JA biosynthesis were also identified, including *ALLENE OXIDE CYCLASE 3* (*AOC3*), which increases, and *LIPOXYGENASE5* (*LOX5* [37];), which decreases during abscission. The presence of key players in the synthesis, perception, signalling and response of ethylene among the candidate genes further supports an important role for ethylene during abscission, provides evidence for possible interactions between ethylene and JA, validates the multi-scaled screening approach and gives weight to the other candidates identified.

Auxin related transcripts were also found common to both ethylene induced and natural abscission. Transcripts included those encoding for proteins involved in auxin homeostasis (CATALASE 2, CAT2.1 and CAT2.2 [38];), and/or transport (PIN-LIKES 6, PILS6 [39];; SNX1 [40];; CLC2 [41];) and conjugation (GH3.5/WES1 [42];) were identified, as well as auxin-dependent transcription (IAA27/PAP2 [43];) and auxin/ubiquitin mediated proteolysis (SKP2A [44];). The transcripts *CAT2.1*, *CAT2.2*, *PILS6*, *SNX1*, *GH3.5/WES1* and *SKP2A* increase in the AZ during abscission, while *CLC2* and *IAA27* decrease. Taken together, these observations provide evidence of the importance of hormone-related transcriptional programs activated during oil palm ripe fruit abscission.

### AZ abscission-specific genes fingerprinted pathways related to senescence, nutritional stress, nutrient recycling, energy and oxidative stress

Our analysis identified genes known to be up-regulated at the onset of senescence, involved in key processes including macromolecule degradation, nutrient salvage and translocation, as well as detoxification and defence. Key senescence markers identified include the *SENESCENCE-ASSOCIATED GENE 15*/*EARLY RESPONSIVE TO DEHYDRATION STRESS 1* (*SAG15*/*ERD1*) gene and the senescence-inducible chloroplast *STAYGREEN1* (*SGR1*) gene [45–47]. Another senescence related gene identified, *AMY1* that encodes ⍺-amylase1, is a stress-induced enzyme secreted extracellularly in Arabidopsis leaves possibly involved in starch degradation after cell death [48]. Key transcriptional regulators known to be directly or indirectly involved in the regulation of senescence including ATAF1, RAP2.4 and MYC2 were also identified [49–51]. The photoassimilate-responsive gene *PAR1*, which encodes a PR-like protein and increases in response to high levels of soluble sugars [52], suggests a metabolic transition from anabolism to catabolism occurs during abscission. Genes were identified that encode glutamate dehydrogenase (GDH), alanine aminotransferase (AlaAT2), 3-methylcrotonyl-CoA carboxylase (MCCB), the alpha-subunit (E1A2) of branched-chain ketoacid dehydrogenase complex (BCKDC) and the Lysine-Ketoglutarate Reductase/Saccharopine Dehydrogenase bifunctional enzyme (LKR/SDH), all key enzymes involved in the catabolism of free amino acids (i.e. alanine, leucine, lysine and glutamate). Markers of nitrogen and phosphate starvation were also found, including a down-regulated major nitrate transporter NRT1 and up-regulated phosphocholine phosphatase PECP1 respectively [53, 54]. In addition, the analysis identified a gene similar to *PYD3*, which encodes beta-ureidopropionase, an enzyme that catalyses a late step in pyrimidine degradation, and functions in the recycling of nitrogen from nucleobases to general nitrogen metabolism [55].

A dramatic switch in energy metabolism during abscission is suggested by the presence of a key BZIP53 TF involved in primary metabolism reprogramming under low energy stress [56]. In addition, genes for enzymes that provide alternative substrates for TCA cycle and oxidative phosphorylation including adenosine monophosphate (AMP) deaminase (*FAC1*) and adenylosuccinate synthetase (*ADSS*) were identified. There were also key glycolytic genes up-regulated, including fructose biphosphate aldolase (*FBA6*) and the phosphofructokinases, *PFK2*, *PFK3* and *PFK5*. In addition, genes encoding pyruvate decarboxylases (*PDC2* and *PDC4*), aldehyde dehydrogenase (*ALDH2B4*) and alcohol dehydrogenase (*ADH1*), key markers of intense glycolytic activity derived towards fermentative metabolism (ethanol and acetate) for energy supply, were also identified.

We found evidence for a major switch in redox control in the AZ during oil palm ripe fruit abscission. Firstly, the transcriptional activation of key players of reactive oxygen species (ROS) homeostasis in peroxisomes were identified including two genes encoding ROS-scavenging catalase homologs to Arabidopsis CAT2, a key mediator of cell redox homeostasis shown to coordinate salicylic acid (SA) repression of auxin accumulation and JA biosynthesis during plant defence [57]. In addition, a gene for a peroxisomal glycolate oxidase (GOX), a photorespiratory enzyme that may also serve as an alternative source for the production of H_2_O_2_ during biotic stress was identified [58]. Secondly, evidence for a key glutathione biosynthetic gene (*Glutamate-cysteine ligase*/*ROOT MERISTEMLESS 1*, *GSH1/RML1*) as well as aldehyde dehydrogenase (*ALDH3H1*) putatively involved in detoxifying aldehydes generated by lipid peroxidation were identified [59, 60]. Thirdly, key markers of adaptation to oxidative stress in plastids were identified including the DnaJ chaperone J8, a nuclear encoded soluble protein found in the chloroplast stroma that accumulates in response oxidative stress, and the stress-induced RELA/SPOT homolog 3 (RSH3, LOC105041854) involved in ppGpp stress signalling in plastids [61, 62]. Finally, evidence for a parallel metabolic adaptation to intense oxidative stress includes the upregulation of transcripts for fructose-bisphosphate aldolase (*FBA6*) and alternative oxidase (*AOX1*), indicative of adaptation to oxidative stress of glycolytic and respiratory activities, respectively [63, 64].

## Discussion

### Common organ abscission genes and processes of eudicots and monocots

Monocot and eudicots phylogenies have evolved mechanisms for fleshy fruit abscission to disperse seeds, but how these mechanisms differ between taxa is unclear. In the current study we used a multi-scale transcriptome analysis to compare the ripe fruit AZ, pedicel, and immature fruit AZ treated with ethylene, AZ during natural abscission and AZ of non-shedding fruit samples, to identify a robust list of core genes and processes that function in the AZ during fleshy fruit abscission of this monocot.

Seed dispersal strategies of higher plants can be divided into three major categories; seed shattering (e.g. domesticated cereal crops of the Poaceae), dry fruit dehiscence (i.e. pod shattering of Arabidopsis and many species of the Brassicaceae and Fabaceae), and fleshy fruit abscission (e.g. tomato as the most studied dicot model [4, 65];). While both seed shattering and fleshy fruit abscission occur through cell separation events in the AZ at the base of the fruit, dry fruit dehiscence involves two cell separation events, in the dehiscent zone (DZ) in the valve margin, and in the AZ at the base of the seed [65–67]. Interestingly, despite anatomical differences between these different dispersal mechanisms, convergent evolution has been observed [67–70]. For example, qSH1 and RPL (REPLUMLESS), orthologs of a BEL1-type homeobox transcription factor, function in the grain AZ of rice and the DZ of Arabidopsis dry fruit respectively [68, 71]. Another example are orthologs of the *Sh1*, which encode YABBY transcription factors that function in the seed AZ of sorghum, rice and maize and were selected in parallel during domestication for the non-seed shattering trait [70]. In the fruit AZ of the oil palm, we found genes that are expressed with similarities to those with known functional roles during dry fruit dehiscence of Arabidopsis, in addition to, *BEL1* (Table 1) with similarity to the those expressed in the seed AZ of the cereals and the DZ of Arabidopsis. This suggests conserved mechanisms of oil palm fruit abscission include those found related to dicot and monocot AZ differentiation, in particular, those related to Arabidopsis dry fruit silique separation (Table 1).

Within the group of candidates we found expressed in the oil palm fleshy fruit AZ, five candidates have similarities to genes that function during Arabidopsis silique dehiscence (*MANNANASE7*, *MAN7*, and *ADPG1*/*PGDZAT*, renamed *EgMAN7*, *EgPGAZ1* and *EgPGAZ2* respectively) and floral organ abscission (*LAC7* and a leucine-rich repeat receptor kinase similar to *HSL1*, renamed *EgLAC7* and *EgHSL1* respectively), which again provides evidence that some established mechanisms of cell separation in the DZ and AZ for model dicots are conserved for this fleshy fruit monocot [30–32, 72]. In particular, we again found key genes for dry fruit dehiscence. For example, *MAN7*, which encodes an endo-beta-mannase, functions in Arabidopsis and *Brassica napus* silique dehiscence, with expression in both vegetative and reproductive organs, with high expression in the Arabidopsis siliques at a stage just prior to the initiation of dehiscence [31, 73]. *ADPG1* also functions in Arabidopsis silique dehiscence, while expression is more specific to the silique DZ and seed AZ, and functions in both processes [30]. These results provide evidence that some cell separation mechanisms in both dry fruit dehiscence and fleshy fruit abscission are conserved, or have evolved in parallel. Interestingly, the combination of mutations in *MAN7* and *ADPG1* increases the indehiscence phenotype, demonstrating the combined function of different classes of cell wall modifiers is necessary for cell separation [30, 31]. Indeed, we identified other transcripts that encode proteins similar to Arabidopsis sequences with functions related to cell wall polymer modifications including those for pectin, lignin, xyloglucan and callose [5, 30, 31, 74–77]. The Arabidopsis LRX4 is involved in cell wall and plant development related to pectin, while the Arabidopsis BXL2 is very similar to BXL1, both of which appear to be involved in polar secretion of pectinaceous mucilage from the seed coat epidermis [74, 75]. In addition, a transcript similar to H[+]-ATPase 2 with possible roles in cell wall acidification mediated by receptor kinase activity was also found [57, 78, 79]. These gene functions correspond closely to processes observed previously in the oil palm ripe fruit AZ during abscission. For example, AZ cells undergo polarized oriented cell wall building activity, which culminates with a decrease in methylesterified homogalacturonan HG (JIM5 signal increases) during abscission in a polarized manner [23]. Furthermore, two gene candidates associated with vesicle trafficking were also identified, which could be involved in auxin related transport, secretion and/or recycling of cell-wall components as found in other abscission systems [80–84]. In addition, changes in the cation environment were also observed in the oil palm AZ during fruit abscission [23], while pH changes are important for AZ function during tomato flower abscission [85]. Finally, a special role of lignin structure in Arabidopsis has been identified [5]. A specific lignin brace structure is found in the AZ cells of the separated organ, and acts to localize cell wall breakdown and spatially limit to abscising cells. In the current study, we found several lignin related transcripts which encode lignin biosynthesis and polymerization enzymes including LAC7, C4H and CBSX-2 (Supplementary Table 3). In Arabidopsis, *LAC7* is expressed specifically in the AZ cells of the separated organ. In the oil palm fruit, we found *LAC7* expression to be especially high in the AZ of ripe fruit that are separating (Fig. 5), which suggests lignin biosynthesis is also important for abscission. In transcriptome study with citrus, lignin biosynthesis transcripts were also found to be expressed specifically in the Starch-rich Area (SA) within the fruit abscission zone (AZ-C), which are also cells undergoing separation [86]. Collectively, lignin appears to be an important feature for a variety of organ abscission contexts, including Arabidopsis floral organs, citrus fruit and oil palm fleshy fruit.

The current study corroborates the importance of pectin related changes during abscission by the identification of two additional PGs (*EgPGAZ1* and *EgPGAZ2*) with similarity to the *Arabidopsis ADPG1* that is essential for silique dehiscence [30]. In addition, a transcript for a pectinesterase (*EgPMEI*) has similar profiles to the three PGs in the AZ during ethylene and naturally induced abscission, with a consistent increase of transcript amounts. Indeed, PG transcripts and activity increase in various species during the abscission process, and can be induced by ethylene or inhibited by auxin [87–92]. In tomato, there is a single PG transcript, pTOM6, expressed during fruit ripening [93–95], while four other PGs (TAPG1, TAPG2, TAPG4, and TAPG5) are expressed in the flower and leaf AZ [89, 90, 96]. In oil palm, *EgPG4* is highly expressed in the mesocarp during ripening and induced in the AZ in response to ethylene [20]. In contrast, *EgAZPG1* and *2* are specific to the AZ (Supplementary Table 6). Similar to *EgPG4*, *EgPMEI* is also expressed in the mesocarp so may also function during mesocarp ripening or provide coordination between the ripening mesocarp and AZ function during abscission. As discussed above, pectin related transcripts encoding beta-xylosidase and a leucine-rich repeat extensin-like protein were also found to be highly expressed during the abscission process suggesting other important pectin modifications involved in cell separation. The main type of pectin in primary walls and the main constituent of the middle lamella is HG, a galacturonic acid polymer that exists in both methylesterified, less methylesterified and unmethylesterified forms [97]. The consequence of PME activity includes not only the demethylesterification of HG, but also the generation of methanol and a decrease in pH [98]. Complete demethylesterified HG increases stiffness in the cell wall due to calcium cross-linking of adjacent HG molecules, while a pH decrease can provide more optimal conditions for hydrolysis by PG. In addition, an increase in demethylesterified HG may be more easily hydrated and lead to cell wall loosening [98]. Indeed, the current model is that PME demethylesterifies HG, which subsequently is more susceptible to PG hydrolysis that could result in cell wall loosening [99]. Finally, combined PME and PG activities could release specific signalling oligogalacturonides, and in the case of the oil palm, could be a potential signal that originates from the primary AZ for activation of cell separation in the adjacent AZs as previously proposed [19].

Hormones, in particular ethylene (promotes) and auxin (inhibits), are well known to be involved in organ abscission, including Arabidopsis floral organ abscission and tomato flower and fruit abscission [4, 100, 101]. For oil palm ripe fruit abscission, both ethylene and auxin are known to function as inducer and inhibitor respectively [19, 20]. In the current study, we found a number of hormone related transcripts, including those related to ethylene, auxin and JA pathways, with differential expression in the AZ during abscission. These results corroborate the importance of these pathways and suggests widespread conservation of function during abscission for these hormones [4].

The IDA–HAE–HSL2 (INFLORESCENCE DEFICIENT IN ABSCISSION- HAESA- HAESA-LIKE2) signalling pathway discovered in Arabidopsis has become a significant focus of research [6, 102, 103]. The pathway involves a peptide ligand-receptor system that consists of the secreted peptide IDA encoded by the IDA gene, and the two leucine-rich repeat (LRR) receptor-like kinases (RLK) including HAE and HSL2. Both *ida* and *hae hsl2* mutants retain their floral organs, while overexpression of the *IDA* gene reverts the *ida* mutant to the wild-type abscission phenotype, and overexpression in the wild-type of either *IDA* or *IDA-LIKE* (*IDL*) gene family members results in early abscission. There is currently a debate whether the IDA–HAE–HSL2 signalling pathway functions in other species and organs such as leaves or fruit. Our previous work provided evidence that the *IDA* and *HSL-like* genes are expressed in the oil palm fruit AZ, and that the IDA peptide enhances cell separation and abscission in the oil palm fruit AZ [104, 105]. In the current study, we show that while a HSL1-like transcript normally decreases in the AZ during both ethylene induced and natural abscission, it is detected at even lower amounts in the non-shedding AZ, which corroborates what was observed previously [104]. This provides further evidence that the IDA–HAE–HSL2 pathway functions in the AZ during oil palm ripe fruit abscission, and appears to be a conserved signalling pathway for both dicot and monocot organ abscission.

### The AZ specific transcriptome contains the transcriptional signature of a large metabolic rerouting associated with a senescence-like program during abscission.

Senescence and organ abscission are both regulated by ethylene, and common transcriptional controls have been found to coordinate the timing of the two process with floral organs of Arabidopsis [106]. The transcriptome survey of the AZ fingerprinted senescence marker genes and specific transcriptional regulators. Among senescence related markers, the *SAG15*/*ERD1* gene encodes the regulatory subunit of the ATP-dependent plant caseinolytic protease (Clp) complex involved in the degradation of accumulated and misfolded chloroplast proteins, while *SGR1*encodes an Mg-dechelatase that represents a first step of the chlorophyll catabolic pathway and a key transcriptional regulation point of senescence-related pigment loss. In addition, the ATAF1 NAC TF serves as a core transcriptional activator of senescence by coupling stress-related signalling with photosynthesis- and senescence-related transcriptional cascades [49]. The RAP2.4 DREB transcription factor plays a key role in modulation of the senescence cascade and controls cell dedifferentiation in response to wounding [50], while MYC2 is a key transcription factor involved in JA-induced senescence processes such as chlorophyll degradation [51]. The transcriptome survey of the AZ also fingerprinted many genes known to be up-regulated at the onset of senescence, i.e. those related to proteolytic and free amino acid catabolic activities, carbon, nitrogen and phosphate salvage and translocation, as well as those involved in alternative routes for energy supply or adaptation to oxidative stress [107].

The identification of certain genes may reflect an intense proteolytic activity in the AZ, either through autophagy or proteasome mediated processes. Indeed, several genes associated with proteolytic activity were identified including a ubiquitin-protein transferase, a RING/U-box superfamily protein, an F-box/RNI-like superfamily protein and a cysteine proteinase superfamily protein. Associated with this senescence-related proteolytic activity, asparagine may be a major transport compound used for organic nitrogen reallocation to other tissues given that a gene for glutamine-dependent asparagine synthase (ASN1) was also identified [108]. Interestingly, phosphate-starvation generally induces reduction of the membrane phosphatidylcholine content to provide an internal phosphate source while replacing membrane phospholipids by galactolipids, a process termed membrane lipid remodelling. Abscission-related phosphocholine phosphatase PECP1 is a major player in this process in Arabidopsis, induced by starvation and involved in the liberation of inorganic phosphate Pi from intracellular sources [109]. In this context, genes for plasma membrane phosphate transporters PHT1 and PHT5 that may facilitate translocation of large Pi amounts released by PECP1 activity towards other tissues were also identified [110, 111]. While FAC1 catalyses the hydrolytic deamination of AMP to IMP (inositol monophosphate) ADSS is involved in the reverse reaction, which suggests a futile purine nucleotide cycle that could produce fumarate, and purine nucleotides that serve as an anapleurotic substrate to fuel the TCA cycle.

A switch in energy metabolism apparently occurs during abscission. In Arabidopsis, the BZIP53 TF promotes dark-induced senescence and participates in the transcriptional reprogramming of amino acid metabolism during the dark-induced starvation response, by triggering accumulation of specific proteins including ASN1 and amino acid catabolic genes. The BZIP53 TF acts downstream of SnRK1 (Snf1-RELATED KINASE1), and specifically coordinates the expression of genes involved in branched-chain amino acid catabolism, which also constitutes an alternative mitochondrial respiratory pathway that is crucial for plant survival during starvation [112]. A similar function in the oil palm fruit could explain the large up-regulation of amino acid catabolic pathways, producing organic acids that may be directly used to funnel TCA and oxidative phosphorylation. In this way, one may note that the energy yield, i.e. ATP production, from branched-chain amino acids (leucine, isoleucine, and valine) and lysine catabolism is particularly high, close to that measured with the oxidation of glucose as a substrate [113]. Furthermore, a futile cycle involving AlaAT and GDH, both detected in the present survey, that could mobilize carbon from alanine toward energy production, has been recently described in plants recovering from low energy stress [114]. Pyruvate produced by the reverse reaction of AlaAT is funnelled to the TCA cycle, while deaminating GDH regenerates, reducing equivalent (NADH) and 2-oxoglutarate to maintain the cycle function.

The transcriptional signatures detected in AZ during abscission for both an active TCA and oxidative phosphorylation as well as fermentative pathway (PDC2, PDC4, ADH1) may be puzzling at a first sight. However, acetate production could be independent of oxygen-limiting conditions, as recently demonstrated in Arabidopsis under drought response where it serves to stimulate the JA signalling pathway to confer stress tolerance [115]. The acetate pathway could also presumably occur under normoxia conditions in the AZ of oil palm fruits. In this way, one may note that ATP-dependant enzymes such as the cytosolic PFK2, the plastidial isoform PFK5, as well as invertase INV1 are transcriptionally induced during abscission rather than their inorganic pyrophosphate-dependent enzyme counterparts, respectively PFP and sucrose synthase, whereas these latter are assumed to be the preferred isoforms under hypoxic conditions [116]. Rather than by oxygen-limiting conditions, induction of the acetate pathway could be hormonally controlled through ethylene and the TF RAP2.2 transcriptional cascade [117].

Overall, how these large metabolic changes are linked to the abscission processes in the AZ are largely unknown, but based on our results, we can conclude that the transcriptional activity in the AZ appear to promote major metabolic transitions both spatially and temporally during ripe fruit abscission. A metabolic transition from anabolism to catabolism before controlled cell death could enable the recycling of major cellular components and nutrient redistribution to other tissues or oxidation towards energy production during the abscission developmental program. Such a program could be triggered by environmental stimuli such as nutrient limitation, i.e. carbon and nitrogen compound shortage caused by phloem flux arrest, or/and by internal hormonal factors (ethylene, JA, ABA). A recent report provided a link between metabolism and the mechanisms of auxin and ethylene regulated abscission [118]. The report demonstrated that auxin regulates sucrose transport during rose petal abscission, antagonistically to ethylene induced abscission.

The ROS pathway functions during leaf abscission of *Capsicum*, floral organ abscission of Arabidopsis, flower abscission of Lupine, and fruit abscission of longan (*Dimocarpus longan*) [119–122]. In the context of the longan fruit, carbohydrate stress induced fruit abscission is mediated by ROS [122]. In our study, we provide evidence that the ROS pathway functions in the AZ during oil palm ripe fruit abscission, and as a possible link between changes in the carbohydrate metabolism during fruit senescence and the abscission process in the AZ. Interestingly, a senescence-like program displaying many genes similar to those detected in oil palm fruit AZ, was observed in Arabidopsis leaves after application 5-chloro-3-methyl-4-nitro-1H-pyrazole, CMNP, a compound known to be a fruit abscission stimulation agent in citrus [123].

### Transcript profiles of a non-shedding oil palm individual substantiate key genes and metabolic processes that function during oil palm fruit abscission.

To validate the functions of candidates identified during ethylene treatment and natural abscission, we examined gene expression in the AZ of a palm that does not abscise its fruit. The AZ in this individual appears to develop normally and the non-abscission character does not appear to be due to a mutation in a gene such as in the tomato *JOINTLESS* or Arabidopsis *BLADE-ON-PETIOLE1/2* [72, 124]. Our transcript profiling results support the hypothesis that the cause of the non-shedding character in this genotype is a change in the regulation of the gene network involved in the abscission process. Furthermore, the results identify processes regulated by the abscission regulatory network during oil palm fruit abscission, given the decreased expression observed in the non-shedding palm AZ. Notably, the abundance of all the pectin related transcripts, which are characterized in having coordinated expression profiles during abscission as discussed above, are all very low in the AZ in the non-shedding palm, corroborating their important function for the abscission process. In addition to pectin related transcripts, many of the other transcripts we identified are either related to metabolism, or defence. One DEG was similar to thaumatin-like proteins, which are pathogenesis-related (PR) proteins with antifungal activity involved in systematically acquired resistance and stress response in plants [125, 126]. Transcripts for thaumatin-like proteins were also found highly expression in the AZ of bean and in the AZ of peach after induction by ethylene [127, 128]. Thaumatin-like proteins may be part of a defensive response by the plant against possible pathogen attack when the separated AZ tissue is exposed to the environment. However, it is unclear whether these proteins may also play an active role in the abscission process. While thaumatin transcripts increase in the mature fruit AZ during both ethylene and natural induced abscission and is low in the non-shedding AZ, an increase is observed in the mesocarp (Supplementary Table 6), so our evidence does not suggest a specific role for abscission, and may carry out defence functions in the AZ and adjacent tissues during and after cell separation that leads to fruit abscission.

## Conclusions

The major objectives of the current study were to identify AZ specific expression related to the timing of oil palm ripe fruit abscission, in order to screen for genes that function in the AZ, and compare to candidates identified from dicot organ abscission models. Through multiscale analyses, we identified a core set of gene candidates with highly coordinated expression in the AZ, some of which have opposite expression in the non-shedding palm fruit AZ. This is consistent with important functional roles for these genes in the AZ that lead to oil palm ripe fruit abscission. Of particular interest, we found a number of previously identified genes mainly from Arabidopsis, including *PGAZ1*, *PGAZ2*, *LAC7*, *MAN7*, *HSL1*, *CBSX1*, *BEL1*, with known functions related to the AZ or DZ during Arabidopsis floral organ abscission and pod dehiscence, respectively. This provides strong evidence for a widespread phylogenetic conservation between monocot and eudicot lineages in the separation zones of both fleshy and dry fruits. The identification of these genes with known abscission and dehiscence functions from model species validates this core set of genes identified with expression closely related spatially and temporally to abscission. In addition, our data points to significant metabolic changes that occur in the AZ during abscission, including key genes and transcriptional regulators related to senescence, nutrient recycling and reallocation, alternative energy supply routes and adaptive responses to oxidative stress. These results open up new perspectives in our understanding of fruit abscission in relation to metabolic changes that take place in the AZ leading up to and during abscission. In addition, the results provide potential gene markers for the selection of elite *E. guineensis* palms with less or delayed fruit abscission.

## Methods

### Plant material

For the ethylene-induced abscission transcriptome analysis, oil palm (*Elaeis guineensis*) fruit bunches at 30 and 150 Days After Pollination (DAP) from a *tenera* clone (clone C) produced in Thailand were collected at the Golden Oil Palm Company Limited, Krabi province as previously reported [20]. For the *E. guineensis* natural abscission and gene candidate validation experiments, AZ samples at 30, 120 and 160 DAP (unripe and no abscission observed in field) and 160 DAP (ripe fruit bunches observed to shed fruit in the field) were collected. For the non-shedding individual and qPCR validation, AZ was sampled from two clones of backcrosses (interspecific hybrid × *E. guineensis*), which show contrasting fruit abscission phenotypes [25]. The first clone underwent normal fruit abscission, while the second clone retained fruit indefinitely (ripe non-shedding, RNS).

### AZ sampling and preparation

For RNA extractions, the fruit bunches were collected and the base of fruits containing the AZ were sampled as follows: the spikelets were removed from the bunches, rinsed in water, then individual fruit containing the base of the fruit containing the AZ were removed with scalpel, then the base of the fruit containing the AZ were dissected (approximately 50 AZ pieces were collected per sample), weighed at approximately 3 g then froze immediately in liquid nitrogen. Backcross material were cleaned and processed with the same procedure total AZ weights were approximately 3–6 g.

### Histology and microscopy analysis

For histology analysis, the base of the fruits containing the AZ were collected and processed as previously described and stained with the following dyes; Toluidine Blue, Ruthidium Red and DAPI for comparison [25]. Samples were then mounted on slides with Mowiol and observed with a bright-field microscope (Leica DM6000) using different objectives (X10, X20, etc. …). To visualize the AZ, tissue sections were also observed and photographs were taken with a Retiga 2000R camera (Qimaging).

### 454 Sequencing data analysis and data mining

AZ (30 DAP and 150 DAP) and pedicel (30 DAP) samples selected for 454 pyrosequencing were obtained by treating spikelets of fruit at 30 DAP and 150 DAP with ethylene at 0 h, 3 h, 6 h and 9 h as previously described [20]. Total RNA from AZ and pedicel tissue samples described above was extracted as previously [129]. The titanium kit (Roche) was used and cDNAs derived from the different tissues and different ethylene treatment time points were tagged independently and then mixed together in one sample for 454 pyrosequencing carried out by the National Center for Genetic Engineering and Biotechnology (BIOTEC), Thailand. The 454 pyrosequence analysis, de novo assembly performed following the methods described previously using an automated pipeline previously described and with support of the SouthGreen Bioinformatics Platform (http://southgreen.cirad.fr/), and the high performance cluster of the Unité Mixte de Recherche Amélioration Génétique et Adaptation des Plantes [130, 131]. After assembled contigs were annotated and cleaned we applied a statistical approach to analyze the transcriptome (Supplementary Fig. 2). The statistical approach was performed as previously with some modifications [131]. Statistical differences in read abundances between the AZ150 DAP (ripe fruit) untreated ethylene field samples (0 h) and each ethylene treatment time point (0 h compared with 3 h, 6 h and 9 h) were identified by Audic and Claverie statistics [132]. A contig was considered differentially expressed during the ethylene treatment time course when it exhibited a highly significant difference in read abundance at *P* = 0.01 when compared to the untreated 0 h sample. The *P* values obtained by the Audic and Claverie test were then adjusted using the Bonferroni correction to control false discovery rate (FDR). The HCA was performed to group the DEGs according to their transcription profile with the tool developed previously ([133];http://rana.lbl.gov/eisen/). DEGs were then statistically compared using Audic and Claverie statistics to the P150 DAP and AZ30 DAP samples to identify ripe fruit (AZ150 DAP) specific or enriched gene expression. Candidates were retained when there was a statistical difference between their expression in the AZ150 DAP samples compared with the P150 DAP and AZ30 DAP for at least one time point (0 h, 3 h, 6 h or 9 h; Supplementary Table 2).

### Illumina data analysis

Sequencing of the three samples (AZ30, AZ120 and AZ160) collected in Thailand was complete by the company GATC Biotech (www.gatc-biotech.com). Low quality reads were removed using Cutadapt. Trimmed reads were mapped using the BWA-MEM package with default parameters [134]. Samtools was used to count mapped reads and the number of reads per kilobase and million reads (RPKM) were then calculated [135]. The oil palm predicted transcripts used for mapping was downloaded from the NCBI website (http://www.ncbi.nlm.nih.gov/, GCF_000442705.1_EG5_rna.fna; January 2015). Annotation of the DEGs with similar profiles during both ethylene and natural abscission was conducted using Mapman (https://mapman.gabipd.org/app/mercator [136];) and expert curation based on recent literature devoted to homologous genes characterized in model plants.

### Primer design and qPCR data analysis

Primer pairs for the statistically significant contig gene candidates were designed using Primer3 and Primer3plus. The designed primers were tested with Amplify application (http://engels.genetics.wisc.edu/amplify) to check primer pair specificity and primer dimer formation. Primers used for this study are listed on (Supplementary Table 4). Primer pairs were tested to estimate the efficiency values based on a standard curve generated from a serial dilution of pooled cDNA (5, 25, 125, 625 and 3125-fold dilutions). Only primers with efficiency values that ranged between 1.8 and 2.0 were retained for qPCR analysis. qPCR was conducted as previous described [25].

## Supplementary Information

**Additional file 1: Supplementary Fig. 1.** Clusters AZ Overview of the four main clusters and multiple sub-clusters found by HCA analysis. Sub-clusters are labelled above the bars, while total number of contigs and percentage of each cluster are indicated at the left end of bars.

**Additional file 2: Supplementary Fig. 2.** (454 seq data Overview of sequencing results from the ethylene treatments)

**Additional file 3: Supplementary Table 1.** (1957 Ethylene AZ150 DEGs annotated and expression summary)

**Additional file 4: Supplementary Table 2.** (502 Tissue comparison DEGs annotated and expression summary)

**Additional file 5; Supplementary Table 3.** (168 NA vs ET DEGs annotated and expression summary)

**Additional file 6: Supplementary Table 4.** (qPCR primers used in studies)

**Additional file 7: Supplementary Table 5.** (23 gene candidates overview of validation and expression)

**Additional file 8: Supplementary Table 6.** (RPKM global for entire transcriptome during Natural Abscission)

## Data Availability

The data discussed in this publication have been deposited in NCBI's Gene Expression Omnibus and are accessible through GEO Series accession number GSE166314 https://www.ncbi.nlm.nih.gov/geo/query/acc.cgi?acc=GSE166314.
