# Supplementary figures and images for: Multi-scale comparative transcriptome analysis reveals key genes and metabolic reprogramming processes associated with oil palm fruit abscission
Source: BMC Plant Biol. 2021 Feb 11;21:92. doi: 10.1186/s12870-021-02874-1 (PMC7879690; doi:10.1186/s12870-021-02874-1)

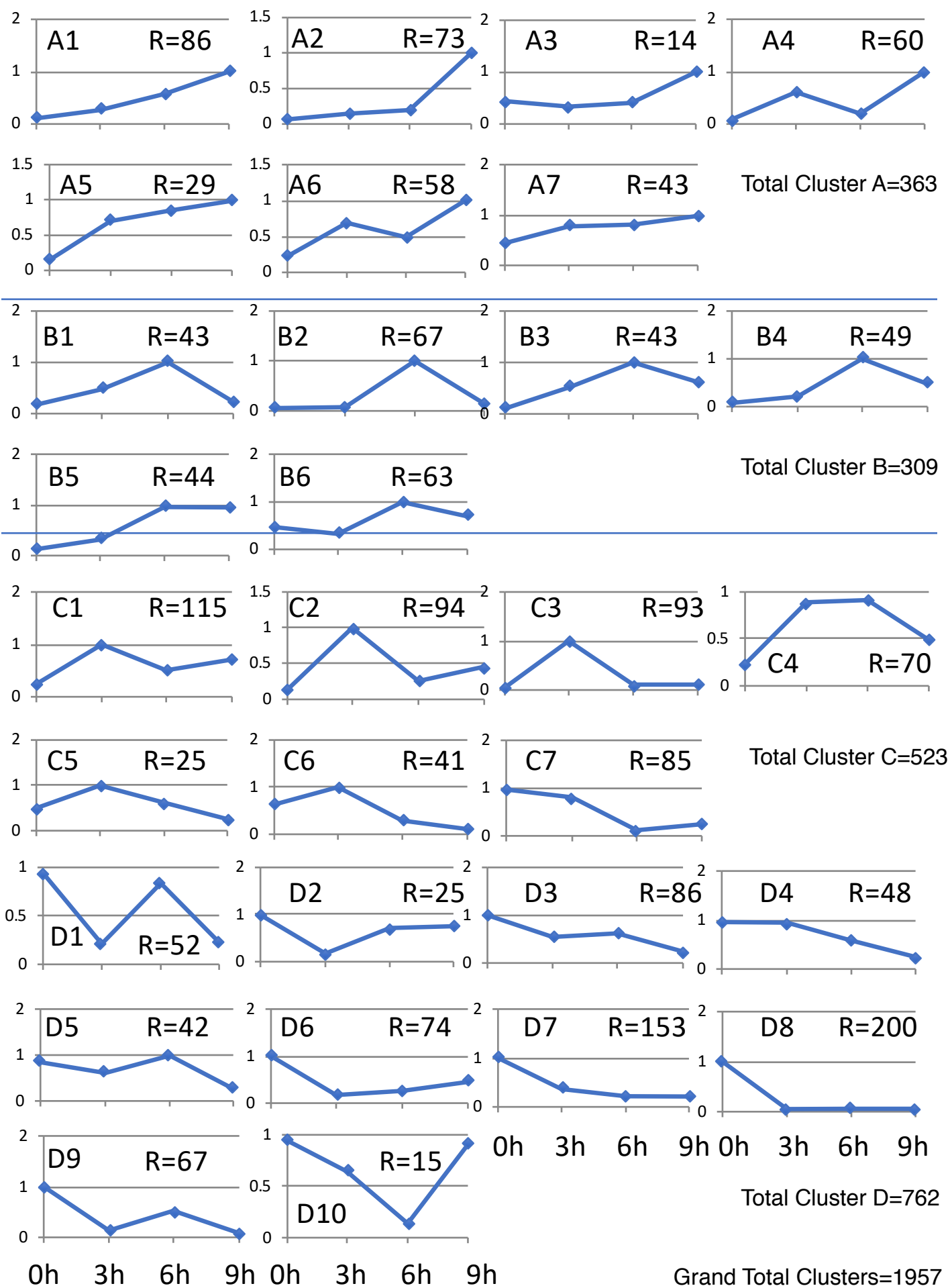

Supplement: Supplementary file 1 — Additional file 1: Supplementary Fig. 1. Clusters AZ Overview of the four main clusters and multiple sub-clusters found by HCA analysis. Sub-clusters are labelled above the bars, while total number of contigs and percentage of each cluster are indicated at the left end of bars. [file 12870_2021_2874_MOESM1_ESM.pdf]
