# Supplementary material for: Multi-scale comparative transcriptome analysis reveals key genes and metabolic reprogramming processes associated with oil palm fruit abscission
Source: BMC Plant Biol. 2021 Feb 11;21:92. doi: 10.1186/s12870-021-02874-1 (PMC7879690; doi:10.1186/s12870-021-02874-1)
Supplement: Supplementary file 2 — Additional file 2: Supplementary Fig. 2. (454 seq data Overview of sequencing results from the ethylene treatments) [file 12870_2021_2874_MOESM2_ESM.pdf]

A

Samples	Number of reads	Avg. length (bp)	Total bp (Mbp)	Subtotals (reads)	Total contigs	Unique contigs
AZ-0h-30DAP	46,048	413	19	657,819	36,583	4,669
AZ-3h-30DAP	164,920	398.2	65			
AZ-6h-30DAP	120,367	422	50			
AZ-9h-30DAP	326,484	425	138.6			
AZ-0h-150DAP	164,027	437	71.6	553,805	33,740	2,856
AZ-3h-150DAP	86,217	371.7	32			
AZ-6h-150DAP	121,711	431.5	52			
AZ-9h-150DAP	181,850	399	72			
P-0h-150DAP	339,657	427	145	1,099,385	40,897	6,930
P-3h-150DAP	278,933	407.8	113			
P-6h-150DAP	416,935	450	187.8			
P-9h-150DAP	63,860	395	25			
**Totals/Averages**	2,311,009	415	971			

B

454 Sequencing data	Numbers
Reads Assembled	1,563,975
Reads not assembled (singletons)	747,034
Percentage singletons	32%
**Total non-redundant contigs**	51,804
